# Pushing the limits: ship rat (*Rattus rattus*) population dynamics across an elevational gradient in response to mast seeding and supplementary feeding

**DOI:** 10.1007/s10530-022-02829-z

**Published:** 2022-06-04

**Authors:** Joanna K. Carpenter, Adrian Monks, John Innes, James Griffiths

**Affiliations:** 1 Manaaki Whenua – Landcare Research, Private Bag 1930, Dunedin, New Zealand; 2 Manaaki Whenua – Landcare Research, Private Bag 3127, Hamilton, New Zealand; 3 Department of Conservation, 18-32 Manners Street, PO Box 10-420, Wellington, New Zealand

**Keywords:** Cold limitation, Resource availability, Density, Pulsed resource, Altitude, New Zealand

## Abstract

Understanding marginal habitat use by invasive species is important for predicting how distributions may change under future climates. We investigated the influence of food availability and temperature on ship rat (*Rattus rattus*) distribution and density across a forested elevational gradient in New Zealand by measuring ship rat demographics following a beech (Nothofagaceae) mass seeding event (‘mast’) at three elevation bands (20–80, 400–500, 800–900 m asl). We tested whether declining food availability limits rat populations at the highest elevation band post-mast by experimentally increasing food abundance above baseline food availability. When our study started 4 months post-seedfall, rats at mid- and low- elevations were at high densities (11.4–16.5 ha^−1^). Rats at higher elevations were barely detectable, but densities peaked (9.4 ha^−1^) 10 months post-seedfall, with the initial increase possibly driven by immigration from lower elevations. All populations declined sharply over the next year. Supplementary feeding at high elevation increased survival, recruitment, and density of rats through winter, 16 months post-seedfall, relative to unfed grids, suggesting food limitation. However, both fed and non-fed populations declined to zero by the following spring, perhaps due to stoat (*Mustela erminea*) predation. Our results suggest that low food availability plays a significant role in restricting rats from cool, high elevation environments. The variation in the timing and magnitude of ship rat responses to the pulsed resource across the gradient also highlights the importance of initial population size and spatial processes as factors modulating ship rat responses to pulsed resources across a landscape.

**Supplementary Information:**

The online version contains supplementary material available at 10.1007/s10530-022-02829-z.

## Introduction

Invasive mammalian predators are a primary driver of global biodiversity loss (McCreless et al. 2016; Doherty et al. 2016). However, the distributions and abundances of invasive species can be patchy in time and space, creating refugia for threatened species (e.g. Olson et al. 2006). Understanding what limits or restricts invasive species within certain habitats is critical for being able to predict whether these refugia will remain, or whether they represent only a temporary reprieve from invasion. For example, warming temperatures under climate change may extend the range of invasive species that are normally limited by cool temperatures, facilitating the invasion of areas that previously functioned as refugia (Bellard et al. 2013; Walker et al. 2019b). The factors that control distributional limits can also vary through time, so under certain conditions invasive species’ ranges may be able to expand before contracting again. For example, the expansion of red foxes (*Vulpes vulpes*) into the Simpson Desert was facilitated by a high rainfall event that created a flush of increased productivity in the system (Sinclair et al. 1998).

Ship rats (*Rattus rattus*, also known as black rats or roof rats) are among the world’s most pervasive and significant invasive species. They are the most widely distributed of all commensal animals (Aplin et al. 2011), and have invaded 50% of the world’s islands (Innes and Russell 2021), where they have contributed to the decline or extinction of many endemic species (Towns et al. 2006; Harper and Bunbury 2015). However, ship rats do not occupy the earth’s highest latitudes, where temperatures are coldest, and they are rare or absent in cool, high elevation habitats (Shiels et al. 2014). For example, they appear to be absent at high elevations (> 3000 m) on Hawai’i Island (winter mean minimum temperature < 2 °C; Amarasekare 1994), and are found at low density near those upper elevations (Amarasekare 1994; Banko et al. 2002). On subantarctic Macquarie Island, their southern limit prior to eradication in 2011, they did not live above 250 m (winter mean minimum temperature 1.6 °C; Pye et al. 1999). In New Zealand, ship rat captures decline with increasing elevation (Christie et al. 2006, 2009, 2017), and they are uncommon or absent in alpine environments (O’Donnell et al. 2017; Foster et al. 2021).

In New Zealand, cooler habitats can act as refugia for rat-sensitive native species (Elliott et al. 2010; Walker et al. 2019b), but the factors that prevent rats from occupying these environments are unclear. One hypothesis is that current ship rat distributions reflect boundaries of the temperatures that ship rat individuals and thus populations can withstand (Shiels et al. 2014). Like all rodents, ship rats have a large surface area relative to their volume, which makes them more susceptible to heat loss than larger mammals (Shiels et al. 2014). In addition, the Indian evolutionary origin of most ship rats (Aplin et al. 2011) suggests they are not cold adapted (compared to Norway rats *Rattus norvegicus* which evolved in north-eastern China). However, relationships between ship rat distribution or abundance and temperature are generally confounded by food availability. Colder, high elevation environments are typically resource poor, so food availability could be the proximate limiting factor at these sites rather than ambient temperature (Shiels et al. 2014; Christie et al. 2017). Understanding what mechanistically prevents ship rats from being common at these sites is important because if ambient temperatures are the primary factor, climate change may have profound effects on the integrity of these refugia.

Ship rats are the most common mammalian predator in New Zealand and have contributed to the local or total elimination of twelve of 30 native forest bird species (Innes et al. 2010). As a consequence, many extant forest bird species are being squeezed into cool, high-elevation forests dominated by beech trees (Nothofagaceae) (Walker et al. 2019a, b). Ship rats are usually found at low densities in New Zealand beech forests, and are uncommon at high elevations (Christie et al. 2017; Walker et al. 2019a; Whitau et al. 2022). Food resources in New Zealand beech forests are pulsed: mass seeding (masting) by the trees every 4–6 years blankets the landscape (e.g. > 2000 seeds per m^2^) in a short-lived resource with cascading effects on invertebrates, birds, and introduced mammals (Kelly et al. 2008). These pulsed resource events provide an opportunity to understand how increased food availability may alter ship rat densities and distributional limits.

Ship rat numbers often go through brief irruptions in response to mast seeding, as the increased resource allows rats to extend their usual summer-autumn breeding season into the winter (Clapperton et al. 2019). They are also detected more frequently at high elevations during mast years (e.g. at 1000 m asl in the upper South Island; Christie et al. 2017). It is unclear to what extent these increases at high elevation are driven by in situ breeding at elevation by small pockets of resident ship rats (which would suggest ship rats can tolerate cool temperatures if they have sufficient food), versus immigration of ship rats from warmer, lower elevations. Top-down pressures may also play a role in regulating ship rat populations in New Zealand’s cool, high elevation forests. Ship rat populations in New Zealand’s warmer podocarp-broadleaf or podocarp-beech-broadleaf forests can be limited by predation by feral cats (*Felis catus,* Efford et al. 2006) and introduced stoats (*Mustela erminea*) (Murphy et al. 2008; Robertson and De Monchy 2012). However, the strength of top-down pressures on ship rat populations is likely to be highly variable in space and time, depending on the relative numbers, fertility and mortality rates of both predators and prey. For example, a ship rat population declined after heavy beech seedfall regardless of whether stoats were controlled (Blackwell et al. 2003), and ship rat numbers did not increase in two 900 ha sites at which stoats were experimentally removed (Ruscoe et al. 2011). Knowledge of when and how stoats suppress ship rat populations across different habitats, including cool, high-elevation beech forests, is still scarce.

Here, we investigate the roles of food and ambient temperature as limiting factors for ship rat populations by measuring ship rat density, survival, recruitment, movement, and demography across an elevational gradient following a pulsed resource event (beech mast) in southern New Zealand. Although the mast event provided abundant food across the site initially, we experimentally supplied supplementary food at high elevations once seed was no longer available to disentangle the roles of food and temperature as limiting factors. We also measured the abundance of predators (stoats) across the elevational gradient through time. We hypothesised that food availability and winter temperatures would be the two key regulators of ship rat density across the landscape at the site, and that this should be apparent through the following mechanisms:

1. Inter-mast ship rat density should be low at all elevations, but especially at high elevations (prediction P1) due to low food availability and colder winter temperatures.
2. The food resource pulse associated with a beech mast (autumn 2019) alleviates ship rat food limitation resulting in higher winter survival, especially at lower elevations, and an extended breeding season from autumn 2019 to summer 2020. This would result in increased densities of ship rats where and when temperatures were not limiting (at low elevations all year, at high elevations from spring 2019 to autumn 2020; P2). Ship rats would have an apparent delayed response to beech seeding at high elevations, compared to low elevations (P3), due to lower initial rat density.
3. Rat survival, breeding and density across the entire elevation gradient would decrease when available food resources diminish once beech seed germinates or rots (from early summer 2020; P4) (Wardle 1984).
4. Winter temperatures would further decrease ship rat survival. Declines would occur earlier at high elevation than mid elevation due to colder temperatures (P5).
5. Supplementary feeding at high elevation would delay the predicted decline in survival, recruitment, and densities until winter temperatures caused the population to crash (P6).

## Methods

### Study system and site

**Fig. 1:**
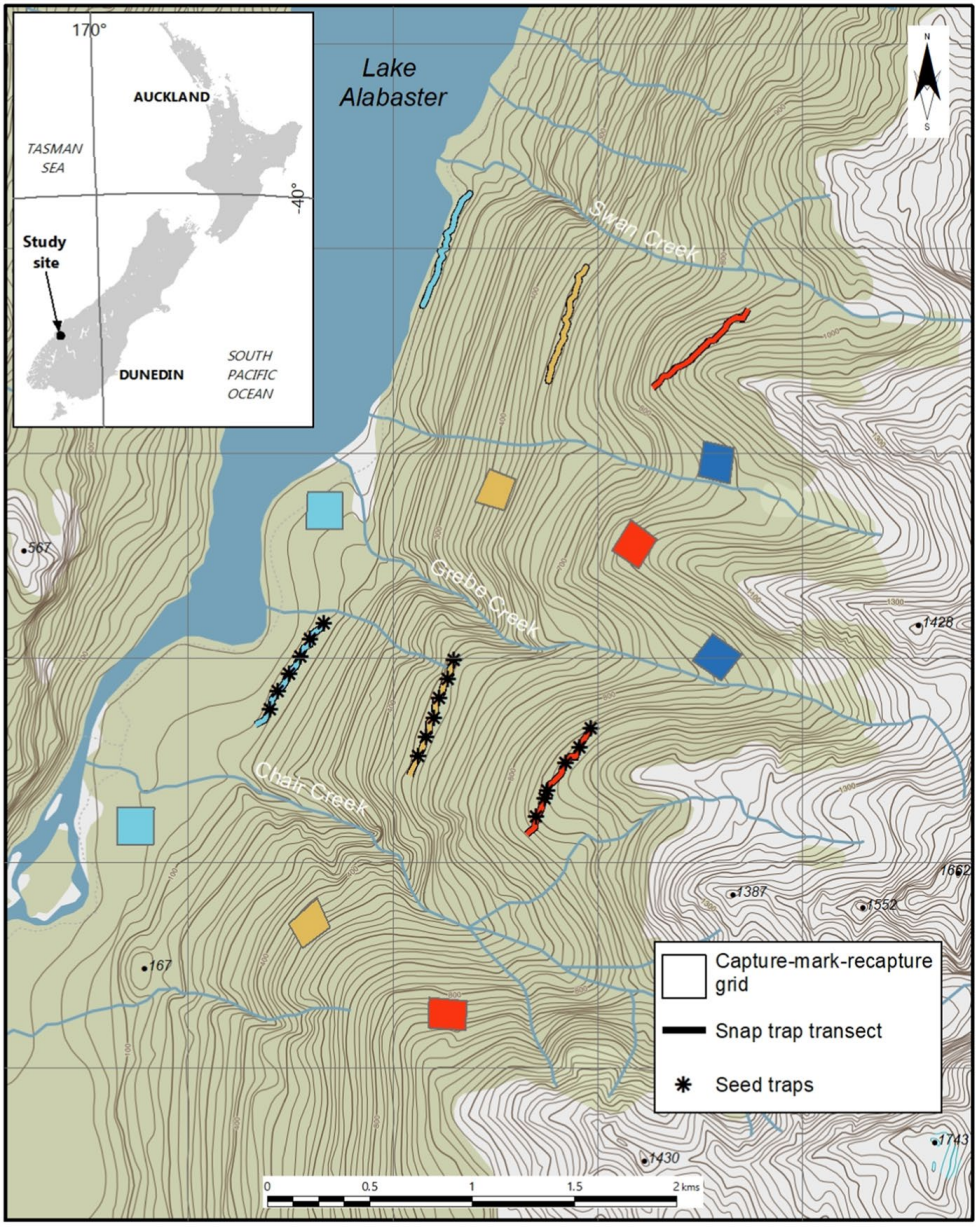
Map showing study site and sampling units, stratified into three elevation bands: low (20–80 m asl, coloured light blue), mid (400–500 m asl, coloured yellow), and high (800–900 m asl, coloured red). Dark blue represents the high elevation grids with supplementary feeding. Snap trap transects also included trail cameras to index stoats and possums and seed traps (marked with black asterisks) to measure seedfall. Grids were 180 × 180 m, and snap trap transects were 600 m long. Green shading represents forest, and white shading represents grasslands and rock

The study site is located in northwest Fiordland, New Zealand, on the eastern side of Lake Alabaster (44.5167° S, 168.1572° E), at the confluence of the Pyke and Hollyford valleys (Fig. 1). The valley floor is approximately 20 m above sea level (asl), and is comprised predominantly of silver (*Lophozonia menziesii*) and red beech (*Fuscospora fusca*), rimu (*Dacrydium cupressinum*), miro (*Prumnopitys ferruginea*), thin-barked totara (*Podocarpus laetus*) and kamahi (*Weinmannia racemosa*) (Mark and Sanderson 1962). At around 500 m this mixed beech-podocarp-kamahi forest grades to species-poor upland silver beech forest, with some southern rata (*Metrosideros umbellata*) and totara (*Podocarpus totara*). The treeline is at approximately 1100 m. The area has a wet, temperate climate with an average rainfall of 4250 mm per year (Ruscoe et al. 2001). The site is bounded by high mountains to the east and a large water body to the west, which probably act as immigration barriers for ship rats.

We stratified the study site into three elevation bands: low (20–80 m asl), mid (400–500 m asl), and high (800–900 m asl), with the mid-elevation sites located just below the threshold where the comparatively species rich forest grades into species poor upland forest. Mean annual temperature is 2–3 degrees lower at high elevation (mean = 6.78 °C) compared with mid elevation (mean = 9.05 °C) and low elevation (mean = 10.1 °C; Suppl Appendix S1). Each elevation band contained two capture-mark-recapture grids (to assess density, survival, and recruitment), and two rodent snap trap lines (to yield carcases for necropsy). Two additional capture-mark-recapture grids were established at high elevation and provided with supplementary food. Trail camera transects to index stoats were established along the snap trap lines, and six seed traps were established on each of three snap trap lines. Capture-mark-recapture grids were at least 300 m away from the snap trap/camera lines. We assume that these were therefore independent as the average ship rat home range size is 150 m in diameter at typical mainland densities (Innes and Russell 2021).

In 2019, the beech trees in the valley masted heavily, producing 3935 silver beech seeds per m^2^ on average based on annual data from seed traps up valley from our site (Suppl Appendix S2). Although these seed traps were checked infrequently so the exact timing of the seedfall is unknown, previous studies have shown that between 70 and 100% of beech seed falls between March and May (Wardle 1984). Our seed traps (established in July following peak seedfall) demonstrated that seedfall remained low following the mast event (between July 2019 and January 2021), with no significant pulses of seed at any elevation (average seeds per m^2^ were 92 at high elevation, 46.7 at mid elevation, and 37 at low elevation; Suppl Appendix S2). Therefore, we conclude that the peak seedfall had happened across the entire elevation gradient by July 2019 when we began monitoring, although peak seedfall at high elevation may have lagged behind low or mid elevation by one or two months (Wardle 1984). Invertebrate biomass was lower at high elevation in spring 2019 and 2020 compared with low- and mid-elevation (Suppl appendix S2). Rat sampling devices were established between May and June 2019, and the first capture-mark-recapture sessions were conducted in winter (July) 2019. Capture-mark-recapture, snap trap, and trail camera data were then collected four times a year, once every season (winter, spring, summer, autumn) until summer (January) 2021. All fieldwork was carried out under a global concession permit (CA-31615-OTH) with the New Zealand Department of Conservation.

### Capture-mark-recapture and supplementary feeding

We estimated ship rat density (ship rats per hectare), survival, and recruitment across time and elevation using spatially explicit capture-mark-recapture. Two capture-mark-recapture hollow grids (hereafter referred to as grids) were used to obtain a ship rat density estimate from each elevation band. Each grid consisted of a 180 × 180 m (3.2 ha) square of 96 metal mesh cage traps (27 × 17 × 13 cm, with a mesh size of 1.5 × 0.5 cm), with 7.5 m spacing between the traps, following the layout recommended by Wilson et al. (Wilson et al. 2007). Each cage trap was covered with a wire A-frame covered in corflute to exclude possums and keep traps dry. For each live trapping session, traps were baited with peanut butter and kept open for 5 consecutive nights, and checked each morning. Grids were run simultaneously. Occasionally, trapping sessions were run for fewer nights due to bad weather. Captured rats were lightly anaesthetised using isoflurane, then a passive integrated transponder (PIT) tag (11.5 × 2.2 mm, Allflex FDX-B glass implantable transponder) was injected under the skin between the shoulder blades and a numbered metal fingerling tag (model 1005–1, National Band and Tag Co.) was attached to the right ear. Each rat was sexed, weighed, and its reproductive status was recorded. We recorded the weight and ID of recaptured rats, then released them. Non-target animals (e.g. stoats or mice) captured in traps were released.

In early summer (December) 2019 we established two new live trap grids at the same elevation as the two high elevation grids, to test the effect of supplementary feeding on rat density, survival, and recruitment. This timing was based on the fact that the beech seed was expected to be unavailable by mid-summer (Wardle 1984) and we wanted to prolong the food pulse. We placed 40 feeding stations (Key Industries Protecta Evo Edge model) on each grid, interspersed between the live-capture traps. The number of rats with access to the stations was approximately 30 per grid, based on the maximum number of animals detected in the January session when feeding began. We used feeding stations to minimise food being eaten by non-target species such as brushtail possums (*Trichosurus vulpecula*), although mice would still have been able to access the food. We ran a capture-mark-recapture survey in early summer (December) 2019 on these two grids to estimate a baseline rat density prior to feeding beginning in midsummer (January) 2020 (the remaining 6 grids already had density estimates from July and October 2019, so these were not run in December 2019). Feeding stations were filled with 30 kg commercial multi-species feed pellets (NRM MultiFeed Nuts) per grid (approximately 750 g per station) on January 13, March 5, May 13, June 13, July 15, August 26, and September 19. We aimed to feed monthly, but this was not always possible because of lockdowns imposed in response to the COVID-19 pandemic. In the first two refilling sessions (March and May 2020), food stations were often empty, but from June 2020 onwards there was usually some food remaining in the stations. Following our December 2019 capture-mark-recapture survey on the fed grids only, we ran capture-mark-recapture surveys on fed grids at the same time as the non-fed grids until spring 2020.

### Reproductive statistics and body condition

To measure ship rat reproductive condition, we established two 600-m-long snap trap transects in each elevation zone to kill rats for necropsy (Fig. 1). Snap trap transects were situated 400–1000 m away from capture-mark-recapture grids. Each transect consisted of 25 Victor snap-traps spaced 25 m apart. Each trap was housed in a plastic tunnel to prevent interference from non-target species. Traps were baited with peanut butter and kept active for 3 nights during each survey. Surveys were generally within 4 weeks of the capture-mark-recapture surveys (i.e., they happened four times a year, once every season), but sometimes later. The number of rats captured each day was recorded and traps were rebaited and reset. Killed rats were removed and frozen until necropsied, and breeding condition and body condition were recorded (see Suppl Appendix S3 for detailed methods and analysis).

### Radio collaring

To determine the fate of ship rats at high elevation once the population had peaked, we radio-collared 39 ship rats at high elevation. We expected that rats would either die in situ, or emigrate downslope. We collared rats > 140 g that were captured in the live traps during capture-mark-recapture sessions between early summer (December) 2019 and autumn (May) 2020 on both fed and non-fed grids. We first anaesthetised rats with isoflurane, then sedated rats before collaring by injecting 0.01 ml Zoletil (made up at half label strength) into the muscle of the hind leg. We fitted each rat with a 7.2 g VHF collar (Holohil RI-2DM) with a mortality signal (which changes the pulse rate of the transmitter if it has been stationary for > 24 h) before releasing them once they had regained consciousness. Following collaring, rats were located from a helicopter every 2 months and the signal (alive or dead) and location were recorded. Once every 3 months we attempted to locate dead rats on foot, ascertain cause of death where possible, and record a more accurate location.

### Predator indexing using trail cameras

We measured stoat relative abundance through time and across the elevation gradient using trail cameras (Gillies 2019). Four trail cameras (either Reconyx PC900 HyperFire, Browning Strike Force HD Pro, Bushnell Trophy Cam Aggressor, or Ltl-Acorn) were placed along each snap trap transect, with 200 m spacing between the cameras. Each trail camera was set 6–20 cm above the ground and focused on a lure of fresh rabbit meat and two pieces of Connovation Erayz paste, pegged to the ground under a mesh cage approximately 1 m away from the device. Cameras were set to take a three-photo ‘burst’ every time an animal was detected, with a 5-min stand down between triggers. Each three-photo burst was counted as one detection. Surveys occurred at the same time as the snap-trap surveys (i.e., four times a year, once every season). We calculated the number of stoat detections per 1000 camera hours over 21 days and used this as an index of stoat abundance and activity (Gillies 2019). Stoat home range size can be highly variable based on habitat and season (King and Veale 2001), and it is possible we detected the same individual stoats across different camera transects, even though transects were spaced a minimum of 500 m apart.

### Analysis of capture-mark-recapture data

We tested predictions P2, P3, and P5 by estimating the population density of ship rats (ha^−1^) across the elevation gradient through time with spatially explicit capture-recapture models (SECR; Efford et al. 2009). We analysed the data from each quarterly capture session in each grid using the ‘secr’ package in program R (Efford 2021a). We assumed that populations were closed during each trapping session (i.e. there was no reproduction, mortality, immigration or emigration during these periods). We fitted spatial detection models, which assume a Poisson spatial point process with a baseline detection rate (*g*_*0*_) and a scale parameter (σ) for the distance function between the estimated ship rat activity centres and the trap locations. For more details on how models were specified, see Suppl Appendix S4.

We fitted five separate models with differing predictors of the density (D) parameter corresponding to four hypotheses: (1) density remains constant across elevations and across surveys (D ~ 1); (2) density varies with elevation but remains the same across surveys (D ~ elevation); (3) density varies across surveys but not across elevation (D ~ survey); (4) density varies with elevation and across surveys (D ~ elevation + survey); and (5) density varies within each survey in a different way for each elevation (D ~ elevation*survey). We expected that if prediction P3 were true, model 5 would be the best supported model. The best model was selected with the Akaike’s Information Criterion corrected for small sample size (AICc) (Burnham and Anderson 2002). Values (ΔAICc) are reported relative to the AICc of the best model, with models of ΔAICc < 2 having substantial support (Burnham and Anderson 2002).

We also used spatial Pradel-Link-Barker open-population capture-recapture models (Efford and Schofield 2020) to estimate survival and recruitment of ship rats between surveys and across elevations, thereby testing prediction P5. We used the ‘openCR’ package in R to implement the models (Efford 2021b).

We first modelled the data from non-fed grids only to see whether survival and recruitment differed across surveys and elevation. Models specified lambda0 (the baseline detection hazard) and σ varying with survey, and survival (Φ) and per capita recruitment (*f*) varying with either survey and/or elevation. We also specified models that allowed between-session movement of home range centres according to a Gaussian dispersal kernel, which reduces bias in survival and recruitment estimates driven by dispersal (Efford and Schofield 2020). The best model was selected using AICc, as detailed above.

Secondly, we tested prediction P6 by testing for differences in survival and recruitment between fed and non-fed high elevation grids separately, using live capture data from January 2020 to September 2020 (encompassing four surveys: summer, autumn, winter, and spring). We ran models with lambda0 varying with survey, and survival and recruitment varying with supplementary feeding and survey. We also specified models that allowed between-session movement of home range centres with a Gaussian dispersal kernel. We kept σ constant for this analysis as σ appeared to stay reasonably constant across time at high elevation. The best model was selected with AICc, as detailed above.

## Results

### Ship rat density

**Fig. 2:**
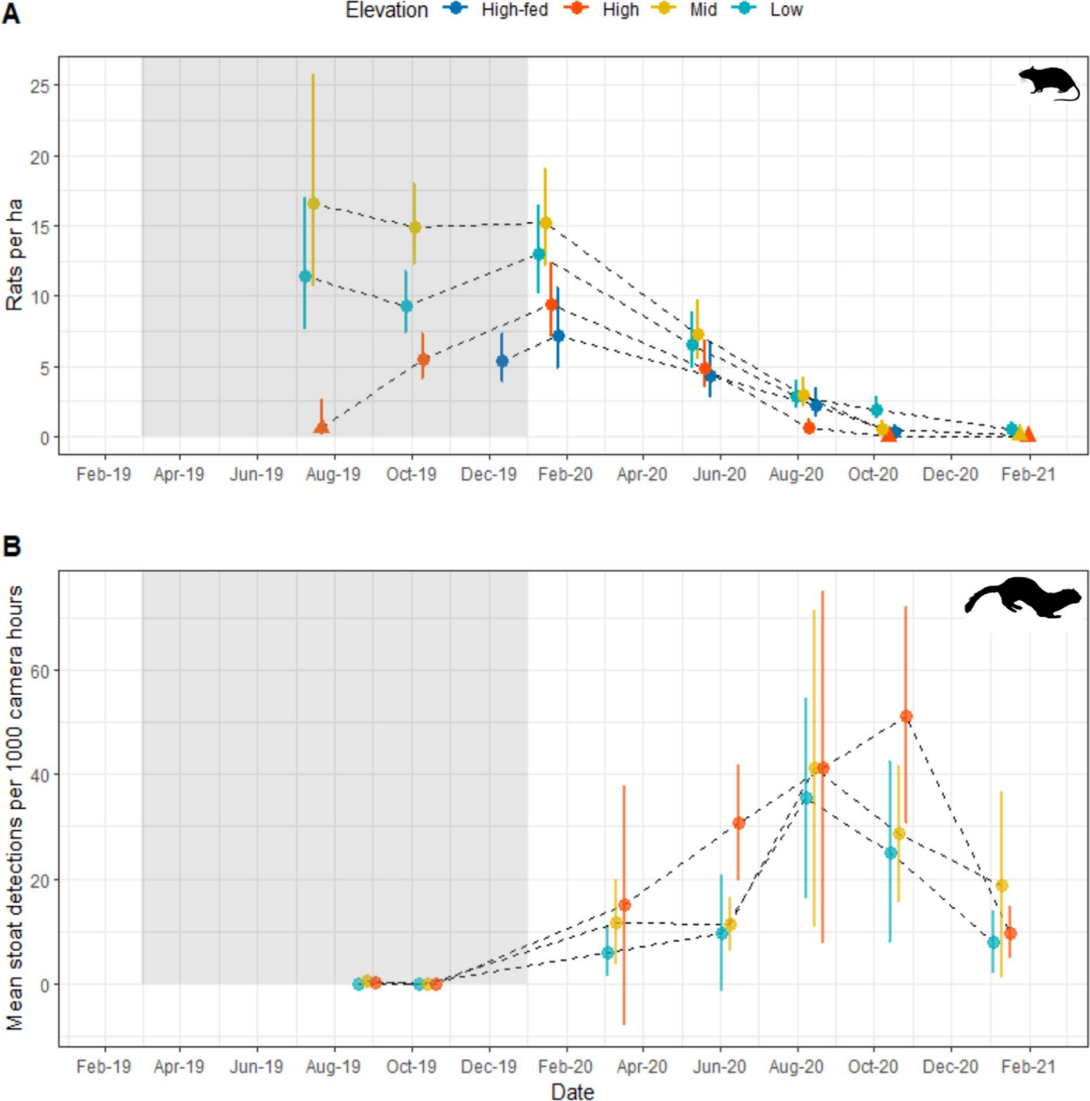
**A** Estimates (95% CIs) of ship rat density across the elevation gradient through time. The grey shading denotes when beech seed was expected to be available to rats, although the seed fell in the autumn of 2019 (following Wardle (1984)). Triangle symbols denote sessions where ≤ 1 rat was captured per grid for that session. x-values have been slightly displaced for clarity. Estimates are pooled across grids within elevation bands. **B** Mean stoat detections per 1000 camera hours 95% CIs) across the elevation gradient

Over the 19 months of the study, we newly tagged 797 ship rats, with 1114 recaptures. Ship rat density (D) varied across elevation and across surveys (Fig. 2A). The model including an interaction between survey period and elevation outperformed the other four models (ΔAICc ≥ 42.76). Ship rat densities already averaged 11.4 and 16.5 rats per ha at low and mid elevations respectively when we began monitoring in July 2019, 4 months after the mast seedfall, supporting prediction P2. In comparison, we captured only one ship rat per grid at high elevation at this time and density was estimated to be 0.6 rats per ha, supporting prediction P3. However, by spring (October) 2019 ship rats were also comparatively abundant (5.4 rats per ha) at high elevations. Densities across the entire elevation gradient declined rapidly between summer (January) 2020 and autumn (May) 2020, supporting P4.

### Ship rat survival and recruitment

**Fig. 3:**
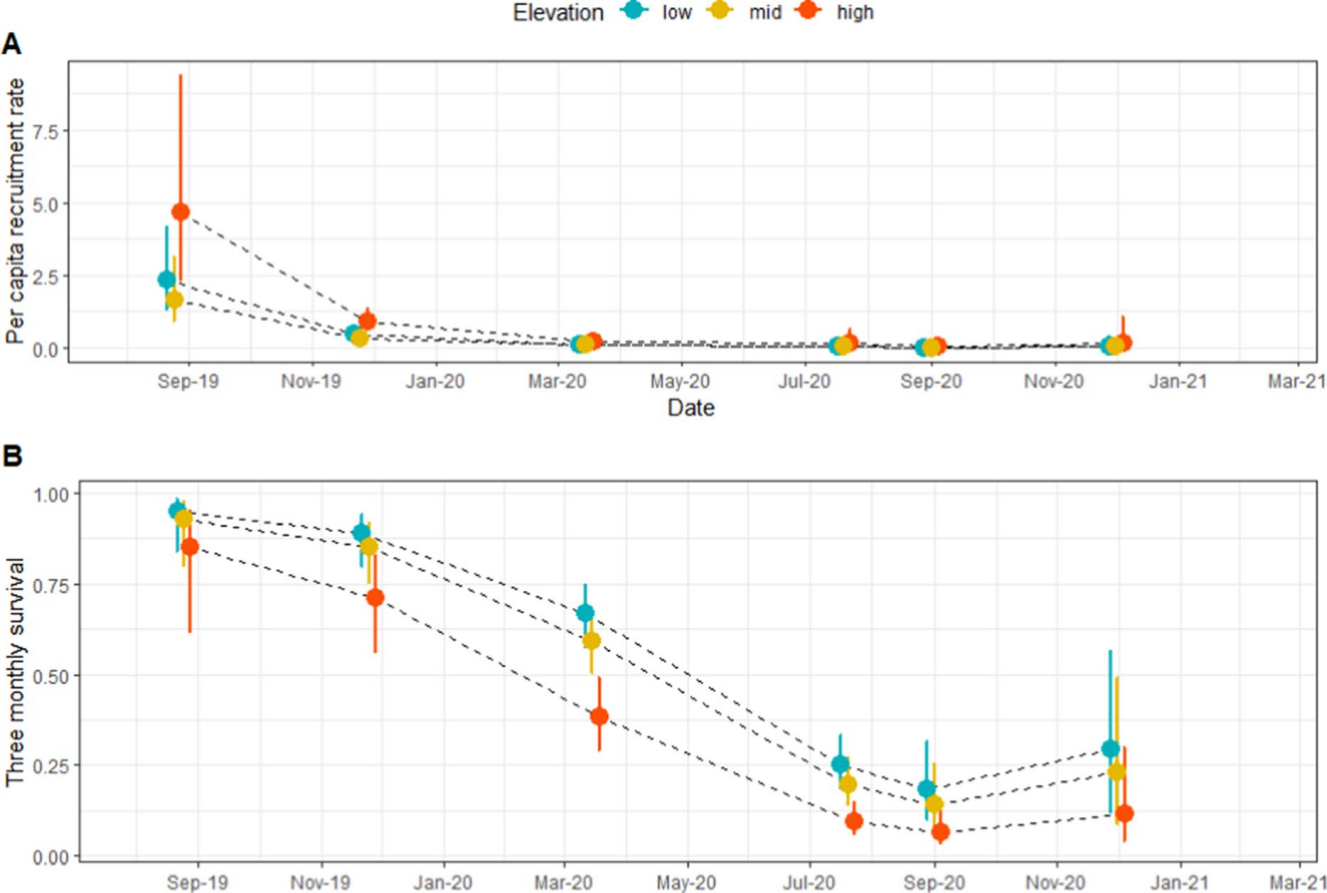
Estimates (95% CIs) of 3-monthly ship rat per capita recruitment (**A**) and survival (**B**) from a spatially explicit open population model where survival and recruitment varied with survey and elevation, and sigma and detection probability varied with survey. Data were from live trapping. Estimates are plotted for the midpoint between two surveys

Our analysis of survival and recruitment (using the capture-mark-recapture data) across the elevation gradient (non-fed grids only) showed that the model with survival and recruitment varying with survey and elevation (but no interaction term between the two factors) fitted the data best (ΔAICc = 17.08). The addition of a bivariate normal movement sub-model that allowed for shifts in ship rat home range centres between sessions did not improve model fit. Recruitment peaked between winter (July) and spring (October) 2019, then declined to near zero from autumn (May) 2020 onwards (Fig. 3a). Between winter 2019 and summer 2020, the greatest rate of per-capita recruitment occurred at high elevation, mirroring the density results that show an approximate nine fold increase in ship rats between winter and spring 2019 at high elevation. Sampling variance could not be estimated for the spring (October) 2020 survey estimates because recruitment was effectively zero. Apparent survival was high across all elevations between winter 2019 and summer 2020, before steadily decreasing until spring 2020 (Fig. 3b), supporting P4 but not P5 (that survival would decline earlier at high elevation due to colder temperatures). As the best fitting model did not include an interaction term between survey and elevation, the model accentuates this pattern.

Reproductive statistics derived from the snap-trapped ship rats (Table 1) also showed that recruitment plummeted in the post-mast year (2020), supporting the live-trapping results and P4. We necropsied 379 ship rats across the study period. Pregnant rats were captured only in 2019. There were sharp decreases in most breeding parameters between winter/spring 2019 (mast year) and winter/spring 2020 (non-mast year) across all elevations, with the exception of the proportion of sexually mature females captured at low elevation, which remained constant. Female rats at mid and low elevations that had previously bred in 2019 had average uterine scar numbers corresponding to two litters, whereas females at high elevation averaged one litter. The increase in uterine scars in females from low- and mid-elevations suggests that these rats had bred over winter, and we also snap-trapped several pregnant rats in winter. Only three ship rats were captured at high elevations in late winter 2019 (giving a mean corrected trap catch of 2.2 rats per 100 trap nights after correcting for captures and sprung traps), but 41 were captured in spring (October and November) 2019 (mean corrected trap catch 18.25 rats per 100 trap nights), mirroring the capture-mark-recapture results. In comparison, the highest trap catch estimate across the study period was at low elevation in November 2019, when we captured 44.4 rats per 100 trap nights. Of these spring captures at high elevation, a minimum of 61% of the females were adults (based on the presence of uterine scars), implying the initial population increase was driven by immigration rather than on-site breeding given their absence at high elevation in late winter. Across all elevations and time periods, males were 60% of captures.

**Table 1:** Breeding parameters of snap-trapped ship rats (*n* = 379)

Parameter	Elevation	2019	2020	
		Winter-spring (maximum density, survival and recruitment)	Summer-autumn (post-peak, declining survival and recruitment)	Winter-spring (poor survival, no recruitment)
Percentage sexually mature females	High	66.7 (18)	71.4 (14)	0 (2)
	Mid	57.1 (28)	47.6 (21)	0 (3)
	Low	28.6 (42)	25 (16)	25 (4)
Percentage females pregnant	High	27.8 (18)	0 (14)	0 (2)
	Mid	3.6 (28)	0 (21)	0 (3)
	Low	2.4 (42)	0 (16)	0 (4)
Mean number uterine scars where present	High	7.9 ± 1.6 (11)	8.9 ± 1.2 (10)	NA (0)
	Mid	15.3 ± 2.3 (16)	12.8 ± 2.2 (10)	NA (0)
	Low	15.8 ± 2.4 (11)	12.8 ± 2.4 (4)	8 (1)
Percentage sexually mature individuals	High	86.4 (44)	74.3 (35)	0 (2)
	Mid	82.2 (73)	66.7 (42)	33.3 (6)
	Low	71.7 (106)	72.5 (51)	57.1 (14)
Percentage sexually mature males	High	100 (26)	76.2 (21)	NA (0)
	Mid	97.8 (45)	85.7 (21)	66.7 (3)
	Low	100 (64)	94.3 (35)	70 (10)

The sample size for each group is shown in brackets

Body condition indices derived from the snap-trapped rats declined with increasing elevation and between 2019 and 2020. A linear mixed effects model where body condition varied by elevation dependent on year fit the data best (ΔAICc ≥ 13.54). Ship rats at low (estimate = 0.076, t value = 3.459) and mid elevations (estimate = 0.033, t value = 1.408) had higher body condition indices than ship rats at high elevation in 2019. In 2020, body condition indices declined across every elevation, but more steeply for ship rats at low and mid elevation compared to high elevation (Suppl Appendix S3).

### Supplementary feeding at high elevation

Supplementary feeding did not make a detectable difference to ship rat density in summer (January) and autumn (May) 2020 (based on overlapping confidence intervals), but in winter (August) 2020 rats were at higher density on the supplementary fed grids compared with the non-fed high grids. Fed rats also had higher survival and recruitment than non-fed, high-grid rats in the three months preceding August 2020 (Fig. 2a). This result was counter to our prediction P6, that supplementary feeding would elevate densities until winter temperatures caused the population to collapse. While the difference in density in winter (August) 2020 was small (2.2 rats per ha on fed grids versus 0.7 rats per ha on the non-fed high grids), this still meant that fed rats at high elevation were at similar densities to rats at low and mid elevation. By spring (October) of 2020, rat density had declined to less than 1 per ha on both fed and non-fed high grids (Fig. 2a), and by summer (February) 2021 we did not detect rats on high non-fed grids (measurement of fed grids ceased in spring 2020).

Using the capture-mark-recapture data, our top ranked model comparing fed versus unfed grids included feeding treatment and time effects on survival and recruitment. The top ranked model also included a movement sub-model that allowed ship rat home ranges to shift between sessions (estimated scale of movement: 20.6 m, 95% CI 10.95, 39.01). This model was only a marginally better fit than the same model without the movement sub-model (ΔAICc = 2.243), but a much better fit than models that did not include the feeding treatment affecting survival and recruitment (ΔAICc = 10.59–12.87). Feeding led to an increase in both survival (β = 0.55 (on logit scale); 95% CI − 0.05, 1.15) and recruitment (β = 24.53; 95% CI 24.17, 24.88). However, survival still decreased over time on fed grids and could not be estimated for spring 2020 due to insufficient data (only three captures, all on one of the fed grids). Between summer (January) and autumn (May) 2020, 3 monthly survival was 0.60 (95% CI 0.44, 0.74) on fed grids versus 0.46 (95% CI 0.35, 0.58) on non-fed grids, and between autumn and winter 2020 survival was 0.26 (95% CI 0.15, 0.41) on fed grids versus 0.17 (95% CI 0.09, 0.29) on non-fed grids.

We examined the fate of individual ship rats at high elevation on both fed and non-fed grids by radio tracking animals every two months, to see whether ship rats died in situ (indicated by the mortality signal) or emigrated downslope post-mast. We collared 24 ship rats on the two fed grids and 15 ship rats on the non-fed high elevation grids between December 2019 and May 2020. Four collars were found slipped, so we treated the total sample size as 35 individuals. Mortality was high across both fed and non-fed grids. All individuals had died by August 2020, and the majority (85.7%) died within 2 months of being collared. We could not estimate the time from collaring to mortality as radio tracking only occurred every two months, so we had no data on when individuals died within that period. One individual was found in a creek bed and had possibly drowned, and seven individuals (20%) were found in stoat dens and were assumed to have been killed by stoats, although they may have died of other causes and then been scavenged by stoats. We recovered another seven bodies (20%) but could not ascertain cause of death, and 18 bodies (51.4%) were unrecovered due to them being in inaccessible locations (mostly deep in holes, but sometimes in tree canopies). The average distance between where rats were collared and where their bodies were detected was 106 m (range 10–575 m), which is within normal ship rat home range size (Innes and Russell 2021). There were no consistent movements downslope that would indicate emigration from high elevations over the period; the maximum elevation loss by any one individual was 100 m. No collared rats were found in a different grid to where they were originally trapped.

### Predator indices

Stoat detections from trail cameras were very low (average of 0–0.49 detections per 1000 camera hours across the three elevations) in the mast year, when ship rats were increasing at high elevation (Fig. 2B). Stoat detections increased considerably in summer 2020 (average of 6–14.9 detections per 1000 camera hours), around the time when ship rats began to decline. Stoat detections remained high for the rest of the study period, although a decline was evident in summer 2021. There appeared to be a positive relationship between stoat detections and elevation, where more stoat detections were recorded at high elevation.

## Discussion

### Ship rat response to the pulsed resource event across the elevation gradient

Our study found significant spatio-temporal variation in ship rat population responses to beech mast across an elevation gradient, which highlights the importance of initial density in facilitating rats to respond rapidly to extra resources. When we began the study, in the winter, 4 months after the usual period of peak beech seedfall (Wardle 1984), ship rats were at high densities at low and mid elevations. Indeed, the density of ship rats at mid elevation at this time (16.5 rats per ha) was one of the highest densities ever recorded on the North and South Islands of New Zealand, although Efford and Hunter (2018) recorded densities of 22 rats per ha following a beech mast in the northern South Island, and ship rats at one North Island site reached 25.8 rats per ha in 2019 (O’Malley et al. 2022). As only two studies have estimated ship rat density in a beech mast year (this one and Efford and Hunter 2018), these densities could be typical. Several pregnant females were snap-trapped during this period, demonstrating that some rats had extended their breeding season into the winter, consistent with other studies on rodent responses to mast events both in New Zealand (King and Moller 1997; Clapperton et al. 2019) and in boreal systems (Wolff 1996). However, at this time, rats were at very low density at high elevations, and only became comparatively abundant three months after the study was initiated (7 months after peak seed fall according to the timing given by Wardle 1984). Between winter and spring 2019 at the high sites the open population modelling gave the highest per capita recruitment rate of all sites and all periods. In addition, most female rats snap-trapped at high elevation in spring 2019 had uterine scars corresponding to one or two litters, demonstrating they were older individuals. We suggest that this high population growth rate, combined with the prevalence of older females in spring that were not present in winter 2019, suggests that immigration was a key driver of the early increase in density. Over a quarter of the female rats snap-trapped at high elevation in the spring of the mast year were pregnant, demonstrating that rats were breeding in this habitat, probably because there was still food available for them (potentially beech seed, invertebrates, or mice). This led to a peak in ship rat density at high elevation in the summer 10 months post-seedfall, whereas populations at low and mid elevation maintained high densities from 4 months post-seedfall through to the summer.

Differences in the magnitude or timing of the pulsed resource across the elevation gradient are unlikely to be the mechanism behind the spatio-temporal variation in ship rat response that we observed. Although Wardle (1984) demonstrated that beech trees have the highest productivity at mid-slope, seedfall data collected in the nearby Hollyford Valley during the first year of our study showed there was high beech seedfall at high elevation too (> 7500 seeds per m^2^ at one 800 m asl seed trap; Suppl Appendix S2). Beech seed does fall later at high elevations compared with low elevations, but this effect is small (lag of one or two months; Wardle 1984), and our seed data demonstrated that no significant seedfall occurred after July 2019. In addition, seedfall data from a nearby site showed that peak silver beech seedfall at moderate to high elevations (520–945 m asl) occurred in April and May (Burrows and Allen 1991), which supports the premise that there was little variability in the timing of seedfall across the elevation gradient. It is possible that seed remained available to rats for longer periods at high elevation due to cold temperatures slowing rotting or germination, but the timing of the rat population decline at high elevation was consistent with declines at other elevations, suggesting this was unlikely.

We recorded higher densities of ship rats at mid elevation compared to low elevation for the first half of the study (although confidence intervals often overlapped), potentially due to higher abundances of beech seed at mid-slope, as discussed above. Conversely, mid elevation may represent a ‘goldilocks zone’, where vegetation is still complex but it is above the winter inversion layer (Norton 1985). However, from October 2020 ship rats had higher densities at low elevation compared to mid elevation, and this relationship persisted until the end of the study, when we estimated densities of 0.52 (95% CI 0.24–1.1) at low elevation, 0.11 (95% CI 0.02–0.82) at mid elevation, and could not detect rats at high elevation. These densities may have been unusually low, although they are in keeping with the only other estimate of ship rat density in beech forest outside a mast year, which was 0.38 rats per ha (95% CI 0.36–0.48) in the nearby Eglinton Valley (Christie et al. 2014). The lack of detection of rats at high elevation is consistent with long-term rat tracking data from the general area (Suppl Appendix S5), which shows that rats are generally only detected at high elevation (> 800 m asl) in mast years.

### What caused the decline?

As we predicted, ship rat survival and density declined steeply from mid-summer (January) 2020 onwards across all elevations. There were three likely interacting drivers of decline at the site: food limitation, cold temperatures, and predation by stoats. The initial decline in survival and density began too early to be related to cold temperature limitation. However, it does correspond to when beech seed germinates (Wardle 1984) and is no longer available to ship rats, supporting the food limitation hypothesis. Body condition of ship rats also declined in 2020, consistent with food limitation. If food limitation was the sole initial driver of the decline, however, we would expect that our supplementary feeding would alleviate this pressure, yet supplementary fed rats still declined at similar rates to unfed rats in late summer. This suggests that either food limitation was not the sole agent of the initial decline, or we did not feed the rats sufficient food to maintain the densities they had reached in the summer.

Stoat predation may have hastened the decline of ship rats at the site, although our study did not explicitly test this hypothesis. Stoats can be active throughout the day and night, and the frequency of occurrence of rats in their diet is positively correlated with rat density (Murphy et al. 1998; King and Veale 2001). Stoat detections spiked around the time that ship rats began to decline, consistent with an influx of young stoats in the system in response to increased rodent numbers (King 1983). Additionally, 20% of the rats we radio-collared at high elevation were recovered dead from stoat dens but could have been scavenged, although the proportion of collared rats killed by stoats may have been much higher as we could not recover > 50% bodies. However, mortality of radio-collared rats appeared much higher than values implied by our estimates of survival from open population modelling, suggesting that radio-collaring negatively impacted survival as found by Theuerkauf et al. (2007). Several other studies have demonstrated ship rats driving bottom up effects on their predators during a pulsed resource event (King 1983; King and Powell 2011), which may be followed by top-down effects later in the cycle, much like small mammal assemblage dynamics in arid Australia (Letnic et al. 2011) and boreal systems (Jedrzejewska and Jedrzejewski 1998). Previous modelling suggests that predators may be able to hasten the decline of a ship rat irruption, and limit low phase populations (Blackwell et al. 2001). However, predators cannot prevent a ship rat irruption if sufficient food supplies exist, mainly because the intrinsic rate of increase of rodents is much higher than that of their predators (Blackwell et al. 2001). More research is needed into how and when stoats may impact rats in both beech- and podocarp-dominated forests (but see Ruscoe et al. 2011 and Whitau et al. 2022).

### Limiting factors for ship rats at high elevation

Our results suggest that ship rat populations can persist at low densities in cool beech forests at low and mid elevations in non-mast years (Fig. 2a), but that these populations increase across the landscape including in marginal, high elevation habitats in response to pulsed resource events. Ship rat populations were at very low density at high elevation early in the mast cycle, and we suggest that their initial increase was predominantly driven by immigration from lower, more favourable habitats. This created a time lag, which may have reduced the magnitude of their response before the food pulse was exhausted. In comparison, ship rats are consistently detected at low and mid elevation even in non-mast years (Suppl Appendix S5), which allows rapid growth to occur immediately in response to the pulsed resource. Similarly, populations of several small mammal species occurred patchily in semiarid habitats in north-central Chile in dry years, but individuals dispersed into other habitats when pulsed resource events occurred in heavy rain years (Milstead et al. 2007). If this pattern is common across cool beech forests, then landscape-scale ship rat control efforts in mast seeding years (Elliott and Kemp 2016) could target populations of ship rats at low and mid elevation before they disperse into upper elevations, thereby increasing efficiency.

We hypothesized that cold winter temperatures are the key environmental filter that restricts ship rat establishment at high elevations in non-mast years by directly impacting survival and recruitment. However, our results suggest that baseline food availability is the main mechanism. With supplementary food, we significantly slowed the rate of decline through the winter months (June–August), which demonstrates that ship rats’ vulnerability to winter temperatures may be offset to some extent if they have sufficient food. Similarly, Howard (1951) found that small rodents can survive freezing temperatures when supplied with sufficient food to maintain body temperature. High elevation beech forests have low floristic diversity, with a sparse understorey and few species that provide annual fruit or seed. Invertebrates are also less abundant in high elevation forests compared to low elevation forests (Suppl Appendix S2; Moeed and Meads 1986) and ship rat fecundity has been correlated with consumption of invertebrates (Sweetapple and Nugent 2007). However, invertebrate abundance and floristic diversity are related to temperature, so cold temperatures could still indirectly limit ship rats by suppressing food availability.

It is unlikely that stoats are preventing the establishment of ship rats in high elevation forests in non-mast years as stoats are typically at very low densities in these forests until rodent irruptions occur (King and McMillan 1982; King 1983), as evidenced by our camera trapping results. However, modelling suggests that it is possible for a low-density stoat population to suppress a low-density rat population (Blackwell et al. 2001).

### Conservation implications

Several endemic forest bird species that are threatened by ship rats are restricted to cool, high elevation forests where ship rat populations are ephemeral and abundance is usually low (Elliott et al. 2010; Whitau 2017; Walker et al. 2019b), and our results support this latter point. Recent bird monitoring in the Hollyford Valley near our study site demonstrated that brown creeper (*Mohoua novaeseelandiae*), rifleman (*Acanthisitta chloris*), kaka (*Nestor meridionalis*), and yellow-crowned kakariki (*Cyanoramphyus auriceps*) detections increased with elevation (Iris Broekema, unpub. data., 2020). This distribution could be a legacy of higher and more persistent rat numbers in the valley floor. Similarly, Elliott et al. (2010) found in another beech-dominated forest site that between the 1980s and 2000s several bird species declined at low elevations but remained stable or increased at high elevations.

However, our study also demonstrates that ship rat populations can reach high densities in cool, high elevation forests when sufficient food exists (e.g., in a mast year), and that these densities exceed impact thresholds for native birds. For example, mohua (*Mohoua ochrocephala*) suffer substantial damage from rat predation when rat tracking rates (the proportion of inked cards tracked by a rat along a 450 m transect) exceed 30%, and for vulnerable orange-fronted kakariki (*Cyanoramphus malherbi*) that threshold is only 5% (Elliott & Kemp, 2016). Concomitant rat tracking monitoring at our site demonstrated that tracking rates at high elevation were between 55 and 100% from when monitoring began in August 2019 until November 2020, when they plummeted to zero (Carpenter unpub. data.).

Additionally, increased rat abundances at high elevation in mast years may have serious indirect effects by subsidising stoats, which are more likely to prey on larger birds such as kea (*Nestor notabilis*). We recorded more stoat detections at high elevation, potentially because mouse activity was also higher at higher elevations (Carpenter, unpub. data), and stoats often prey on mice (Jones et al. 2011). Foster et al. (2021) also recorded a positive relationship between stoat detections and elevation in dryland environments, although they largely attributed this distribution to competition with cats and ferrets (*Mustela furo*). As cats and ferrets are extremely rare at our site, we think that the pattern we observed is either related to prey availability, or is an artefact of different detection probabilities across the elevation gradient, e.g. stoats at high elevation were hungrier so were more likely to interact with lures.

Increased beech seed production (Allen et al. 2014) and warmer temperatures leading to higher invertebrate abundance will increase the resource available in high elevation habitats for ship rats, and may lead to permanent establishment of ship rats at higher elevations (e.g. see Harris et al. 2022). In turn, this could lead to more spillover of ship rats into alpine environments, with deleterious consequences for the sensitive flora and fauna that currently survive there (O’Donnell et al. 2017). Ship rats are already occasionally captured above the treeline in Fiordland and Mount Aspiring National Parks (O’Donnell et al. 2017; McAulay et al. 2021). We recommend that further research be undertaken to understand whether immigration is the key process behind ship rat increases in high elevation environments. If so, then experimental control of source populations could prevent spillover into other sites.

## Supplementary Information

Below is the link to the electronic supplementary material.

Supplementary file1 (DOCX 190 KB)
