# Supplementary material for: Pushing the limits: ship rat (Rattus rattus) population dynamics across an elevational gradient in response to mast seeding and supplementary feeding
Source: Biol Invasions. 2022 Jun 4;24(10):3065–81. doi: 10.1007/s10530-022-02829-z (PMC9166931; doi:10.1007/s10530-022-02829-z)
Supplement: Supplementary file 1 — Supplementary file1 (DOCX 190 KB) [file 10530_2022_2829_MOESM1_ESM.docx]

Appendices for:

Pushing the limits: Ship rat (*Rattus rattus*) population dynamics across an elevational gradient in response to mast seeding and supplementary feeding

*Biological Invasions*

Joanna K. Carpenter*, Adrian Monks, John Innes, James Griffiths

*Author for correspondence.

[carpenterj@landcareresearch.co.nz](mailto:carpenterj@landcareresearch.co.nz)

+64 27 747 8778

**Appendix 1**

*Temperature*

We measured temperature over an annual cycle at different elevations at the site using Onset HOBO dataloggers (UA-001-64 pendant model). We attached a datalogger to a metal stake 1.3 m above the ground on each of the six original (i.e. not supplementary fed) mark-recapture grids. Dataloggers were programmed to record the temperature once every 15 minutes. We established the dataloggers in winter (July) 2019, but due to some technical problems with logging data we present data from autumn (May) 2020 until autumn (May) 2021.

The mean temperature from May 2020 to May 2021 was 2–3 degrees lower at high elevation (mean = 6.78°C) compared with mid elevation (mean = 9.05°C) and low elevation (mean = 10.1°C). In the winter months (June–August), the mean temperature at high elevation was 4.25°C, compared with 6.42°C at mid elevation and 6.58°C at low elevation (Fig. 1). The coldest month at high elevation was September 2020, with a mean of 3.18°C. Generally, mid elevation grids were also colder than low elevation grids, except in early winter (May–July 2020), when temperatures at low elevation grids were similar to mid elevation grids.


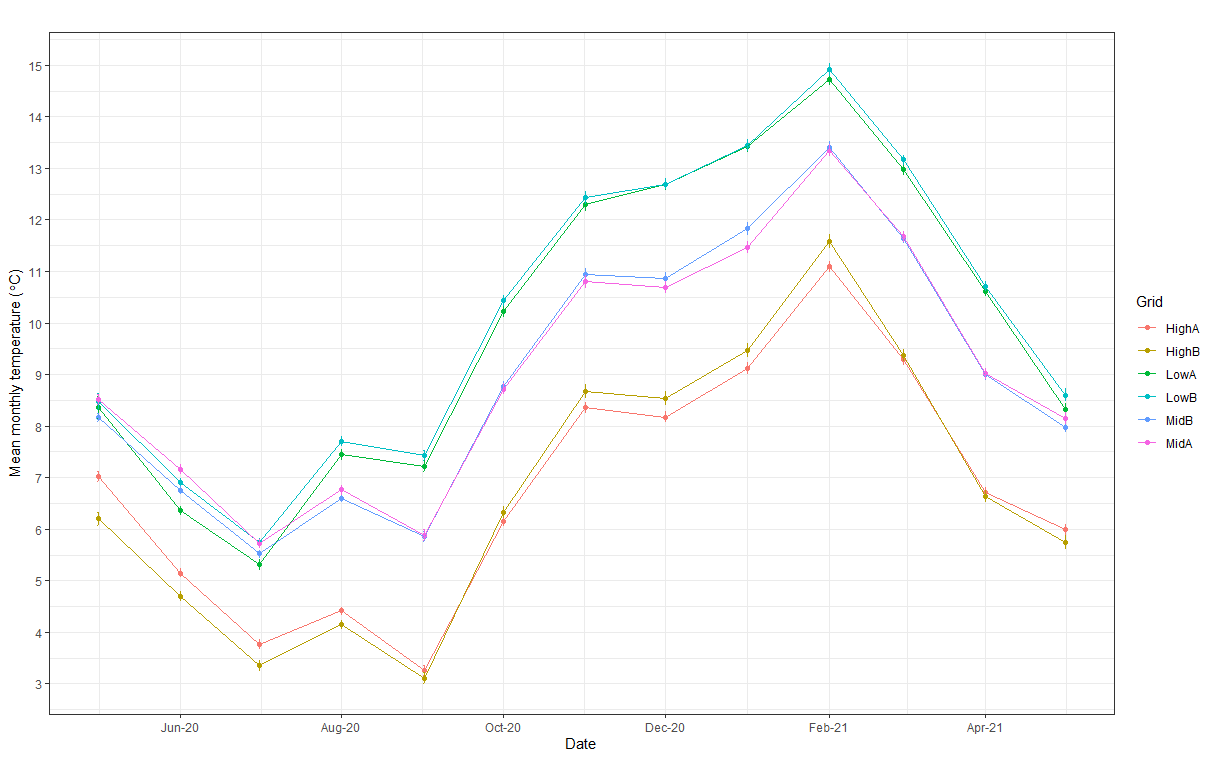


Fig 1: Mean monthly temperature measured from May 2020 to May 2021 across the six non-fed live capture grids. Error bars are 95% CI.

**Appendix 2**

*Food abundance indices – invertebrate biomass*

We estimated the abundance of litter-dwelling invertebrates, an important food for ship rats (Innes & Russell 2021), across the elevation gradient in spring 2019 and 2020 using pitfall traps. Spring is the period during which invertebrates comprise the greatest part of the diet (Innes, 1979). Two transects consisting of five pitfall traps, each spaced 5 m apart, were established in each elevation band. Each pitfall trap consisted of a 100-mm-deep plastic cup (105 mm diameter) containing 50 ml of 50% propylene glycol and 15 g salt, with a wooden lid suspended above to keep rain out. Traps were set in September of each year and were cleared 4–8 weeks later. Invertebrates with body lengths greater than 5 mm were sorted from each sample, oven dried at 60°C for 24–72 hours, and weighed. We only used the dry weight of invertebrates >5 mm as this is the minimum prey size known to be consumed by ship rats in New Zealand (Best, 1969; Innes, 1979). We fit linear mixed effect models to test for differences in the availability of invertebrates for ship rats (indexed by the mass of invertebrates caught in pitfall traps) between elevation bands. We used ‘transect’ as a random effect to account for non-independence between pitfall traps on a transect, with year (2019 or 2020) and elevation (low, mid, high) as a fixed effect. We log-transformed the response variable (dry weight of invertebrates per pitfall trap) to account for non-normality. AICc was used to determine which model provided the most support for the data, out of three possible models: 1) invertebrate biomass ~ elevation + year + (1|transect), 2) invertebrate biomass ~ elevation + (1|transect), invertebrate biomass ~ year + (1|transect).

There were fewer litter-dwelling invertebrates at high elevation compared with mid and low elevation in spring 2019 and 2020. The model that included both year and elevation as predictors of invertebrate biomass was better supported than models that only included year (ΔAICc = 3.5) or elevation (ΔAICc = 4.1) as predictors. The average dry weight of invertebrates per pitfall trap (averaged across both years) was 0.08 g (SD = 0.17) at high elevation, compared with 0.21 g (SD = 0.22) and 0.34 g (SD = 0.68) at mid and low elevation, respectively. Pitfall traps were left open for different durations in 2019 and 2020, so we cannot tell if there were more invertebrates in either year.

*Food abundance indices – seedfall*

We measured seedfall across the elevation gradient, to ascertain whether any seed fell after the initial mast event. Six seed traps placed at 100 m intervals were established on one of the snap trap lines within each elevation band. Each seed trap consisted of a mesh funnel with a catching area of 0.2 m^2^, supported on a metal tripod. Seed traps were located to sample forest-level seedfall rather than the seedfall from individual trees. Seed traps were cleared every 3 months and the samples were dried and sorted. Seeds and fruits were removed from each sample, identified to species, and counted. Seed traps were established in July (after the peak seedfall period). We therefore used annual data from 20 seed traps established by the New Zealand Department of Conservation further up the Hollyford valley between 88 and 839 m a.s.l. to estimate the size of the beech mast event in 2019. Although these seed traps were several kilometres away from our study site, we expected them to represent the general conditions at our site.

Using annual data collected from seed traps up valley from our site, the average seedfall for 2019 was 3935 silver beech seeds per m^2^, indicating a moderate mast year (Fig. 2). There was high seedfall at mid and high elevation in particular (Fig. 3). Our seed traps (established in July following peak seedfall) demonstrated that seedfall remained low following the mast event (between July 2019 and January 2021), with no further significant pulses of seed at any elevation. Seed trap samples were dominated by silver beech seeds (99% of total seeds), with very occasional red beech and pigeonwood (*Hedycarya arborea*). The average number of seeds collected per m^2^ at high elevation was 92 (SD = 139), compared with 46.7 (SD = 126) and 37 (SD = 85.2) at mid and low elevation, respectively.


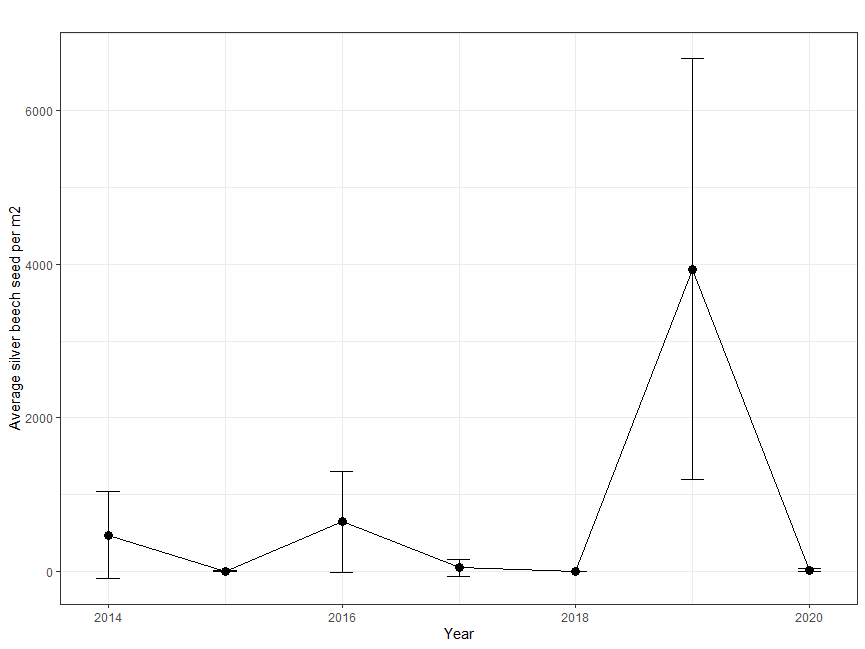


Fig 2: Mean silver beech seedfall per m^2^ captured in the Hollyford Valley from 2014 to 2020 (DOC unpublished data). Error bars are standard deviation.


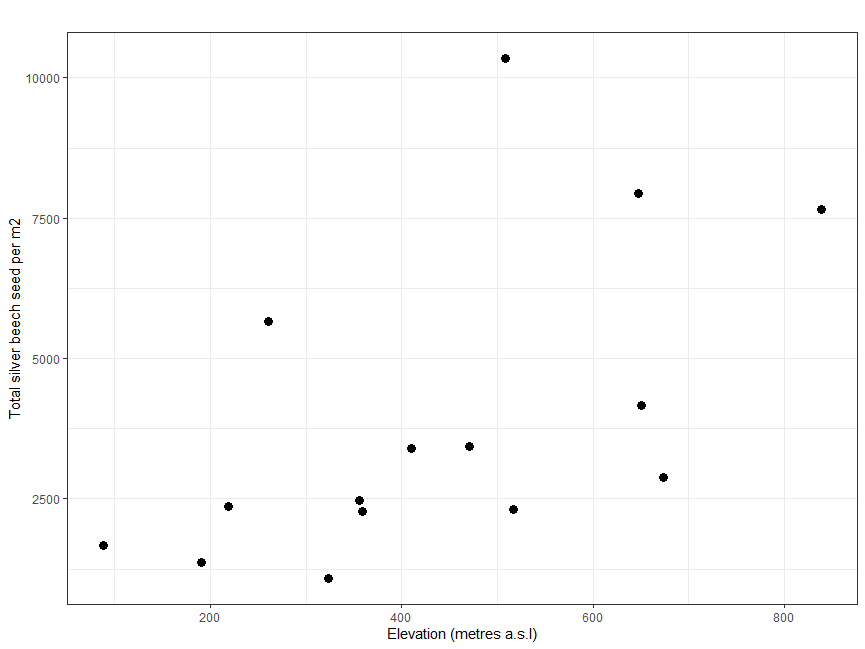


Fig 3: Silver beech fall plotted against elevation, captured in the Hollyford Valley in 2019 (DOC unpublished data).

**Appendix 3**

*Breeding statistics and body condition*

We recorded whole body weight, head-body-length (HBL), and tail length of each rat, omitting rats that had been chewed in the trap or were missing appendages. We did not age the rats using the toothwear index developed by Karnoukhova (1971) as this method is laborious and Efford et al. (2006) cast doubt on its relationship with known-age ship rats. Rather, we identified sexually mature individuals. Sexually mature females were defined as those that were pregnant or had uterine scars, as per Clapperton et al. (2019). Male breeding condition was determined by whether the tubules in the epididymis were visible, although we also recorded whether testes were in the abdominal or scrotal position. We used the following index of body condition for each rat: body condition index = body mass/(HBL^3^)x10^5^ (Moors, 1985).

We used the package ‘lme4’ (Bates et al., 2015) to fit linear mixed effect models to test for the effect of elevation and year (mast year 2019, or post-mast year 2020) on the ship rat body condition index, derived from the necropsied animals. We set survey as a random effect and used AICc to determine the best model out of four models: 1) body condition ~ elevation*year + (1|Survey), 2) body condition ~ elevation + year + (1|Survey), 3) body condition ~ elevation + (1|Survey), and 4) body condition ~ year + (1|Survey).


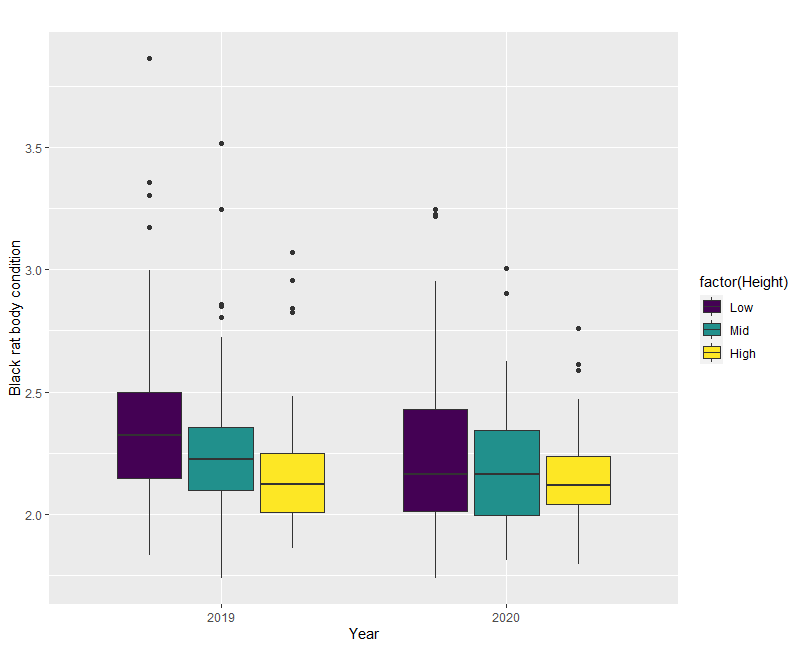
Fig 4. Box plots of ship rat body condition by elevation, for 2019 (mast year) and 2020 (post-mast year).

**Appendix 4**

*SECR models*

We fit SECR models as follows. The detection function followed a half-normal curve, and we set the spatial buffer around each grid at 180 m after examining the scale parameter. We maximised the full likelihood to fit models, but as maximum likelihood estimators do not exist for single-catch traps, we set the trap type to multi-catch. Previous studies have shown that estimates are robust to this kind of model misspecification (Efford, Borchers, & Byrom, 2009). We treated traps that were sprung empty or caught non-target species as not set for that night.

We specified models with *g*_0_ (the probability of capture per day in a trap located at the centre of the home range) allowed to vary with ‘phase’ and elevation (as a factor; low, mid, high, high-fed), as we expected all of these covariates to influence detection probability. For the phase factor, we treated each survey as a different factor level until autumn 2020, when we pooled the following three surveys (winter and spring 2020, summer 2021) together as one factor level due to very low detections. We did not include an effect of a learned response on *g*_0_ as preliminary modelling showed this did not improve model fit. We specified σ to vary with phase as previous studies on rodents have shown their spatial scale of movement to vary across time in mast and non-mast years (Bogdziewicz, Zwolak, Redosh, Rychlik, & Crone, 2016).

**Appendix 5**

*Regional rat tracking data and results*

We examined long-term tracking data collected by the Department of Conservation from a 25-km radius around the study site to test whether ship rats were always present at low elevation. Landscape scale aerial poison operations occurred in 2017 and 2019. Rats are always detected at low elevation (14–200 m a.s.l) except for 6 months after the poison operation occurred in 2017. However, our study site was not treated with poison, so rats are assumed to have still been consistently present.


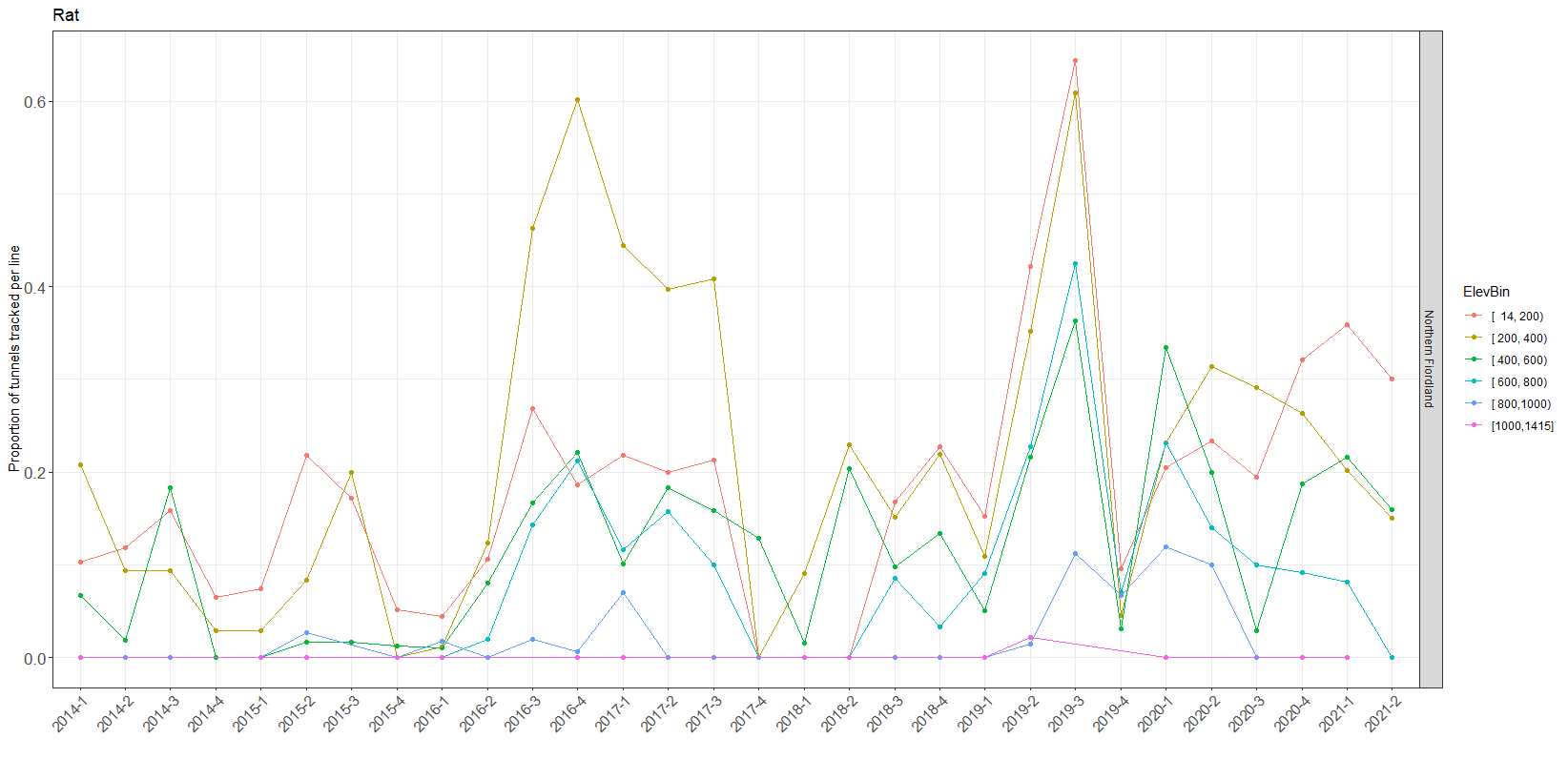


Fig 5. Ship rat tracking results by elevational bin, for a 25-km radius around the study site. Aerial poison operations targeting rats occurred in 2017 and 2019.

**References:**

Bates, D., Maechler, M., Bolker, B., Walker, S. (2015). Fitting Linear Mixed-Effects Models Using lme4. Journal of Statistical Software, 67(1), 1-48. doi:10.18637/jss.v067.i01.

Best, L. W. (1969). Food of the roof-rat, Rattus rattus rattus (L), in two forest areas of New Zealand. New Zealand Journal of Science, 12, 258–267.

Bogdziewicz, Michał, Zwolak, R., Redosh, L., Rychlik, L., & Crone, E. E. (2016). Negative effects of density on space use of small mammals differ with the phase of the masting-induced population cycle. Ecology and Evolution, 6(23), 8423–8430. doi: 10.1002/ece3.2513

Clapperton, B. K., Maddigan, F., Chinn, W., & Murphy, E. C. (2019). Diet, population structure and breeding of Rattus rattus L. In South Island beech forest. New Zealand Journal of Ecology, 43(2). doi: 10.20417/nzjecol.43.22

Efford, Murray G., Borchers, D. L., & Byrom, A. E. (2009). Density estimation by spatially explicit capture–recapture: likelihood-based methods. In Modeling Demographic Processes in Marked Populations (pp. 255–269). doi: 10.1007/978-0-387-78151-8_11

Innes, J. (1979). Diet and reproduction of ship rats in the northern Tararuas. New Zealand Journal of Ecology, 2, 85–93.

Karnoukhova, N. G. (1971). Age determination of brown and black rats. Soviet Journal of Ecology, 2, 144–147. Retrieved from https://scholar.google.co.nz/scholar?hl=en&as_sdt=0%2C5&q=Age+determination+of+brown+and+black+rats&btnG=

Moors, P. J. (1985). Norway rats (Rattus norvegicus) on the Noises and Motukawao Islands, Hauraki Gulf, New Zealand. New Zealand Journal of Ecology, 8, 37–54. Retrieved from https://about.jstor.org/terms
